# Factors that support children and young people to express their views and to have them heard in healthcare: An inductive qualitative content analysis

**DOI:** 10.1177/13674935241258515

**Published:** 2024-06-04

**Authors:** Clare Davies, Donna Waters, Jennifer Fraser

**Affiliations:** 1 Susan Wakil School of Nursing and Midwifery, Faculty of Medicine and Health, 4334 The University of Sydney, Sydney, NSW, Australia

**Keywords:** Children’s rights, children’s participation, decision-making

## Abstract

Despite development of healthcare charters supporting Article 12 of The United Nations Convention on the Rights of the Child, children and young people remain largely silenced in discussions about their healthcare. This article is based on the premise that children and young people should be able to exercise their right to express their views and be heard in all matters that affect their lives. This study examined children’s and young people’s experiences of expressing their views and having them heard in an Australian healthcare context. Using child-centred inquiry and ‘draw, write, and tell’ methods, data were collected from 20 children and young people. Five factors that supported children and young people to express their views and have their views heard were identified: time, relationships with health professionals, communication, teamwork, and family support. By paying attention to these factors, clinicians and others in health settings can better facilitate child-centred practices and support children and young people to express their views and have those views heard.

## Introduction

The United Nations Convention on the Rights of the Child (UNCRC) is an international human rights treaty designed to protect children around the world. Article 12 of the UNCRC states that:

“*State Parties shall assure to the child who is capable of forming his or her own views the right to express those views freely in all matters affecting the child, the views of the child being given due weight in accordance with the age and maturity of the child.”*(UNCRC, 1989)

While the UNCRC defines children as those below the age of 18, it also recognises that there are clear differences in children’s capability in accordance with their age. In recognition of these differences, the term young people is used in this article to describe those between the ages of 12 and 18 years.

Healthcare charters in numerous countries align with principles outlined in the UNCRC (Children’s Healthcare Australasia, 2010; Hospital, 1998), but children and young people still experience challenges in having their views heard in healthcare (Davies et al., 2022; Kilkelly and Donnelly, 2011). Known advantages of children’s participation in healthcare decision-making include feeling valued and respected (Coad et al., 2008), better treatment engagement (Bejarano et al., 2015), and reduced anxiety and fear (Coyne and Gallagher, 2011; Gilljam et al., 2016).

Recent research has identified children and young people wish to have a say in their care (Coyne and Gallagher, 2011; Foster et al., 2022), yet their exclusion from decision-making processes remains widespread (Coyne et al., 2014; Kilkelly and Donnelly, 2011; Lundberg et al., 2021). Healthcare decision-making and service planning largely excludes the perspectives of those under 18 years, with a persistent perception that children lack capacity to make decisions in their own best interests (Davies et al., 2022). The views of children are often disregarded in service evaluations, while parents’ opinions are consistently given close consideration (Nordlind et al., 2022; Starlight Children’s Foundation and Children’s Healthcare Australasia, 2021).

Cultural and social constructions of childhood place children as vulnerable and in need of adult protection (James and Prout, 2015; Johnson and West, 2018). These beliefs shape children’s status in society and influence their right to have a say in what happens to them, including healthcare settings (Ford et al., 2018). These notions of childhood started to be questioned with the rise of postmodern social movements like feminism, and a new paradigm for the study of childhood emerged (Holt, 1975). Referred to as the ‘new sociology of childhood’, this paradigm questioned notions of childhood as a stage in ‘becoming’ an adult. Rather childhood is viewed as a state of ‘being’ (a part of society, not a pre-condition to it). Children can thus be seen as social agents shaping their own lives as well as the societies in which they live, able to competently participate in society (James and Prout, 2015).

Over the past two generations, a shift has occurred in the way children’s healthcare is delivered in developed countries (Jolley and Shields, 2009). Family is seen as a key determinant of well-being for sick children, and family-centred care has become embedded in paediatric care practices of many countries (Shields, 2010; O' Connor et al., 2019). However, family-centred care can place parents at the heart of care delivery, leaving children as passive recipients (O' Connor et al., 2019). More recently, care provision has been reframed through a child-centred lens, placing children at the centre of care delivery (Coyne et al., 2016a; Ford et al., 2018), and an appreciation that listening to children’s perspectives is essential to the delivery of child-centred care (Söderbäck et al., 2011).

Healthcare providers may share traditional beliefs about children’s competency and ability to make informed decisions, especially when a child has a serious illness (Coyne et al., 2014). Protecting children is a powerful motivator but can lead to creation of boundaries around children that exclude them (James et al., 2007).

Although research attention has been paid to participation of children in healthcare decision-making, there is little research that specifically focuses on Article 12 of the UNCRC. Article 12 not only includes the right to participation but also the right to be engaged, heard, and taken seriously (White, 2020). Research that investigates children’s and young people’s experiences of expressing their views and having those views heard in healthcare is required (Davies et al., 2022).

### Aim

The aim of this study was to understand children’s and young people’s (from here on referred to as children) experiences of their rights to express their views and to have those views heard in healthcare. The research questions were a) What are children’s experiences of expressing their views in healthcare? and b) Do children feel their views are heard and taken seriously?

## Research Design

### Methods

Child-centred inquiry was employed in this study, a form of critical research that upholds child rights and places children’s voices as central to the research process (Clark, 2011b) Child-centred inquiry is grounded in social constructivism, assuming that existing ideas about children’s abilities are socially constructed. It challenges assumptions that children are incapable of forming and expressing reliable, valid information about their lives (Clark, 2011a).

### Setting and sampling

Patients in two tertiary children’s hospitals and in the child and adolescent unit of a large general hospital in Sydney were invited to participate. Children under the age of 7 years were excluded from this study as inclusion of younger children would have required different data collection methods, and this was outside the scope of this study.

It was important that participants had sufficient experience of healthcare for a comprehensive understanding of the topic being studied, and therefore, children who had experienced frequent hospitalisation or visits to ambulatory care services (three or more admissions/visits per year) were included. Aboriginal and Torres Strait Islander children were respectfully excluded from this study, as the authors did not have cultural authority to undertake research with and about Aboriginal and Torres Strait Islander peoples, or inclusion of a First Nations researcher on the team to facilitate this. Inclusion and exclusion criteria are outlined in Table 1.

**Table 1.** Inclusion and exclusion criteria.

Inclusion criteria	Exclusion criteria
Children and young people between 7 and 18 years of age, who	Children and young people less than 7 or greater than 18 years of age, or children who
• were current patients of any of the study hospitals, and	• had experienced infrequent admissions and/or visits to ambulatory services, or
• had experienced numerous admissions to hospital and/or visits to ambulatory services, and	• did not speak English, or
• were English speaking	• were seriously ill, or
	• who identified as Aboriginal or Torres Strait Islander

Children and their families were informed about the study through the clinical governance units of the participating hospitals, clinical nurse consultants, nurse unit managers, and an advertising flyer distributed in relevant wards and clinic waiting areas. A letter and age-appropriate participant information was sent to children and parents who had a history of three or more admissions or outpatient visits in the prior 12 months. Children expressing interest in the study were invited with their parents to attend an individual appointment for a more in-depth discussion of the research study.

In keeping with the methodological principle that children can understand and participate in decision-making processes, informed consent for inclusion in the study and recording of ‘tell’ sessions was obtained from all young people over the age of 12 years, and assent was obtained from children under 12 years with the addition of parental consent. The Sydney Children’s Hospitals Network Human Research Ethics Committee approved the study (LNR/18/SCHN/115).

### Data collection

Understanding children’s experiences from their point of view is a key tenet of child-centred inquiry. Researchers therefore have a responsibility to provide children with opportunities to be listened to in safe, inclusive, and engaging ways (Lundy and McEvoy, 2012). One way to achieve this is by using arts-based data collection methods (Carter and Ford, 2014; Driessnack and Furukawa, 2012). Drawing, painting, storytelling, and collage were used to help facilitate children to express themselves (Carter and Ford, 2013).

The data collection method used was ‘draw, write, and tell’ (Angell et al., 2015) which involves children producing artwork or writing a story to help them describe an experience or phenomenon. This is followed by the child being asked to describe their artwork and its meaning. It is important that the researcher does not try to interpret the artwork themselves, as this is seen as a subjective adult interpretation of a child’s experience (Angell et al., 2015).

Children were invited to individual sessions and given a range of materials to create artwork or a story that described their experiences of expressing their views and having their views heard in healthcare. In individual follow-up ‘tell’ sessions, children were asked to discuss their artwork, explain its meaning, and discuss their experiences. These ‘tell’ sessions were conducted by the lead author who had experience of using ‘draw, write, and tell’ as a research method. Parents were invited to sessions if this was desired by the child. ‘Draw, write, and tell’ sessions were conducted in the health services where the child was currently a patient. ‘Tell’ sessions were recorded and field notes were taken. Audio files were de-identified, transcribed by a paid transcription service, and stored in a secure, university-approved database.

### Data analysis

Qualitative content analysis was undertaken by the lead author. In data analysis, as in society, children’s views are at risk of being overpowered by those of adults, and therefore, interpretive analysis methods can be problematic. Qualitative content analysis is a ‘systematic and objective means of describing and quantifying phenomena’ (Elo et al., 2014). Inductive and deductive approaches can be used, depending on the purpose of the research (Elo and Kyngäs, 2008). Deductive content analysis is undertaken to test existing theory, while inductive content analysis adopts an open-minded approach, allowing understanding to emerge from the data. Using both approaches allows for incorporation of different types of data into the analysis process, thereby providing a more complete understanding of the phenomena being studied (Armat et al., 2018; Graneheim et al., 2017). Elo and Kyngäs checklist for establishing trustworthiness in qualitative content analysis was used (Elo et al., 2014). The results of a deductive qualitative content analysis of data from this study are published in Davies et al. (2024). This paper reports on results from an inductive content analysis, identifying factors that children say support them in expressing their views.

**Figure 1.**
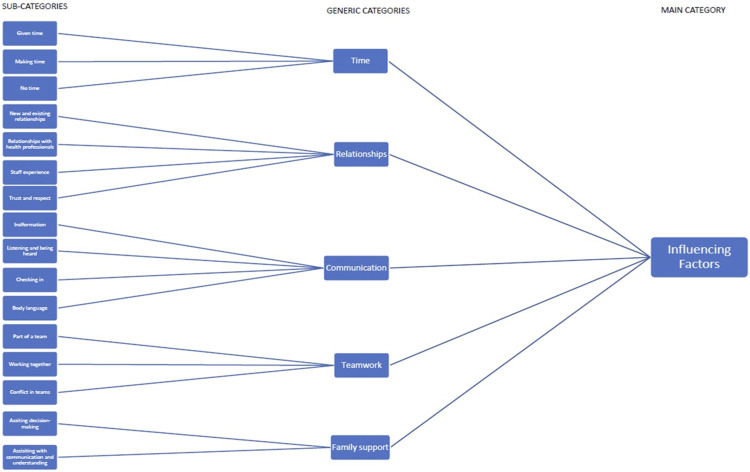
Generic category descriptions.

Data were subject to abstraction, a process of forming a general description of the research topic, and involved generating main, generic, and sub-categories using content-characteristic words (Elo and Kyngäs, 2008).Open codes were assigned to sections of the text that described features in the data; then guided by these codes, text was structured into sub-categories. Sub-categories were named and assembled into generic and main categories (Figure 1). During the data preparation and organisation phases, NVivo Version 12 (QSR International Pty Ltd).

## Findings

Twenty children and young people aged 7 to 18 years (male = 12, female = 8) were recruited to the study. Data saturation is not a concept applicable to qualitative content analysis, and this number was seen to be appropriate for the scope of the study. No participants who consented to be part of the study pulled out. While unintentional, most participants were over the age of 11 years old (*n* = 11). Two participants had transitioned to adult services and therefore had experienced both paediatric and adult healthcare. It was recognised that this would likely result in different perspectives from these participants. Data collection took place from July 2018 to December 2019 and ‘tell’ sessions ranged from 20 to 60 min. Pseudonyms have been used in this article.

Five generic categories were identified that facilitated children in expressing their views and having their views heard. These categories were time, relationships, communication, teamwork, and family support.

### Time

Time was an important factor in children having their views heard and was referred to throughout the ‘tell’ sessions.

Children described how some health professionals ‘made time’ to listen. These health professionals would show an interest in them and their lives and make genuine attempts to understand their experiences. Children acknowledged that health professionals were busy, and that time was in short supply. Staff that made time to listen, to explain in a way children understood, and to ‘*listen to me as a person’* (Participant 1), were described as helpful, as welcoming, and as making children feel included. Other children described how health professionals ‘gave them time’ to ask questions, process information, make decisions, and have a say, especially during consultations.

*“Well often times you know they come into my room, maybe they tell me something different and they’ve given me a substantial amount of time, you know, to process it … the information.”* (Callum, age 16)

As previously outlined, children understood staff were busy, and this was a major obstacle to being listened to. Children described how staff would have to attend to other patients, leaving no time for conversations. They also described being rushed during clinic appointments, which were largely focused on test results and treatment plans, leaving no opportunity for children to be involved in decision-making.

*“I didn’t feel very comfortable there; it was very in and out … they just say, ‘Okay you come’, they test my sugars, they test my HbA1c, told my parents and then kicked me straight out. Like nothing about how’s it going….”* (Charlie, age 16)

### Relationships

Relationships were identified as a key factor in children feeling supported, encouraged, comforted, and safe. Children described having particularly close relationships with nurses, especially those who had known them for many years. Nursing staff were described as understanding, helpful, and friendly. Children described how nurses spend the most time with them and therefore were well-positioned to build strong relationships. These relationships were key to children feeling listened to and heard. Medical staff were described as having less time to build these relationships.

*“I trust all of them (the nurses. They are all kind.”* (Chloe, age 8)

In contrast, children referred to the burden of continuously having to build new relationships in healthcare. This continuing need to build relationships with new staff members led to frustration. Constant rotation of junior staff was highlighted as a key issue in not being listened to and heard. Junior medical staff were perceived as more interested in following processes than listening to their experiences. As a result, they were less likely to include children’s views in care planning. Children reasoned that those who have little experience of working with children did not fully appreciate how they might understand their own illness.

*“They might be in that residency for about six weeks or they might be longer … and at the beginning maybe they’re seeing you as a patient, not you as a person.”* (Anna, age 18)

Staff who were experienced in caring for children were described as better at listening and taking their views seriously. Health professionals who did not have experience of caring for children often ignored their views in favour of parents, as described by one participant:

*“The ‘adult’ doctors, well a lot of them want to talk to the parents a lot more than you which is a bit frustrating sometimes.”* (Vicki, age 16)

Close relationships with health professionals that children had known for years led to feelings of respect, seen as essential for harbouring trust. Feelings of disrespect occurred when children were excluded from conversations, when adults failed to credit their understanding of their illness, or when their feelings were dismissed.

*“It’s not nice to leave people out … That’s just disrespectful. It’s just disrespectful not to listen.”* (Daniel, age 12)

### Communication

Children in this study expressed a strong desire for open and honest communication with adults, highlighting the importance of having their opinions listened to and respected on all topics, not just those related to their healthcare.

Essential communication skills for health professionals included being able to communicate information regarding the child’s illness and care. Children did not want information to be kept from them and fundamental to this was their ability to receive information in a way that they understood, including the need to moderate language and explaining care in age-appropriate language.

*“All the child needs is a bit of explaining about what decisions they should choose and which is best for them.”* (Benjamin, age 13)

Children referred to times when they felt excluded from conversations and to health professionals listening and directing conversations at their parents rather than them. This left them feeling of being treated ‘like a child’, particularly when they felt adults were speaking for them.

*“I’m happy with that, as long as they listen and talk to me…then that’s fine.”* (Rebecca, age 14)

Good communication included staff ‘checking in’ with children, making sure that they had given their consent to treatment and procedures, and not just assuming they were okay with what health professionals were doing. It also included staff regularly asking if they needed anything and showing concern about their welfare.

*“Like tell the new people that like if there is something that’s hurting the person, the like person that’s getting the operation, like stop and ask them what’s wrong.”* (Michael, age 7)

Some participants described the importance of adults identifying their emotions through their body language and react accordingly. One participant described how staff knew her so well that they would be able to decipher her feelings without her having to say anything, and she recognised this as a skill that had been developed from many years of caring for children. This was seen as a particularly important skill, particularly when caring for children who were not verbal.

*“Because children are not always reacting in the same way as adults do, so it’s about those skills and those abilities to read body language and to read other ways of communication.”* (Anna, age 18)

### Teamwork

Children identified shared teamworking as a key factor in achieving optimal health outcomes. This included listening to one another, exchanging ideas, and making collective decisions. They identified that they had responsibility when it came to the team and that they needed to work with health professionals and their parents.

*“I think it’s in between all of our responsibilities basically, I mean we’ve all sort of got to work together to fix it.”* (Rebecca, age 14)

Being involved in team processes such as nursing hand-over and multi-disciplinary team meetings helped children feel valued and gave them a sense of ‘everyone working together’.

*“Yeah so, it’s kind of like that clinical meeting in the morning but the patient’s involved and it was really good and really beneficial … everyone was still able to hear the patient’s opinion in that aspect … so it’s like a handover but the patient’s involved, which I think is really good.”* (Anna, age 18)

There were times when children did not feel part of a team. Some describe how their personal autonomy could be disregarded and how decisions were made based on what was seen as ‘best for them’. Their wishes would sometimes conflict with their parents’ wishes, and decision-making would default to their parents in these circumstances. Children described feeling ‘in the middle’ of conversations between parents and health professionals, almost like onlookers rather than participants, and how, when his happened, their views could be ignored.

### Family support

Understandably, parents and carers were important in helping children express their views during healthcare encounters, and this was demonstrated in several ways. When it came to making decisions, children generally asked for help from their parents. Sometimes children felt comfortable in leaving decision-making to their parents, particularly when there were difficult decisions to be made or when they were younger.

*“Sometimes I just sort of, and I sort of get worked up about it and then it just takes me ages. I still get included, but she sort of helps make the bigger decisions.”* (Rebecca, age 14)

Children also expressed how parents helped them understand information. Communication with health professionals could sometimes be challenging, especially when they were stressed or worried, and they would turn to their parents to help them understand and express their opinions.

*“They wouldn’t look at my parents, they would ask me questions, sometimes I’m like confused, I’m like ‘Mum, can you help me answer these questions?”* (Ruby, age 17)

Families were therefore very important in helping children to express their views, to be heard, and to be taken seriously. However, on occasions, children felt limited by their parents, especially when they talked on their behalf. Some talked about wanting to be more independent and speak for themselves, wanting to be heard and have a say, and wanting their parents to respect their autonomy. Children appreciated that parents wanted to protect them but felt they underestimated their ability to understand and make decisions.

## Discussion

While children’s health charters clearly support the premise of Article 12, that children have a right to express their views and be heard on all matters that impact their lives, children and young people continue to experience challenges in doing so (Davies et al., 2022). This study aimed to investigate children’s experiences of expressing their views and having their views heard in healthcare, and an inductive qualitative content analysis identified factors that children said supported them to have their views heard and taken seriously.

Time was recognised as an essential enabler in children being able to express their views and being heard. This study identified that when adults take time to listen, children feel valued and respected. Other studies have identified time as essential to children’s inclusion in healthcare consultations and their care, with a lack of time being a major inhibitor (Kiili et al., 2021; Traseira and Singh, 2020). When time is limited, children can be denied opportunities to express their views and opinions and therefore be heard. Health services are often time poor and children in this study recognised time a precious commodity. Children need time to understand, express themselves effectively, and build relationships, but lack of time continues to be a major inhibitor of children’s ability to be heard in healthcare (Starlight Children’s Foundation and Children’s Healthcare Australasia, 2021).

Relationships with health professionals that foster trust and respect are also essential in assisting children to express their views (Foster et al., 2022; Kebbe et al., 2019; Lundberg et al., 2021) and for participation in decision-making (Bjønness et al., 2020; Teleman et al., 2021). A recent Australian study found that these positive relationships were fostered when staff listened to them and ‘checked in’ on them (Foster et al., 2022). These findings were replicated in this study, which also found that interactions with inexperienced or disinterested individuals can leave children feeling disrespected, particularly when they do not receive recognition of capability or are invited to contribute to conversations about their illness or treatment. Children often build these close relationships with nurses (Kilkelly and Donnelly, 2006). This study highlighted how nurses were the most supportive health professionals and play an essential role in facilitating children to express their views and have them heard.

Health professionals who are skilled and effective at communicating with children of any age, and who can impart information in a way that children understand, are critical to children being able to express their views and have those views heard in healthcare (Lundberg et al., 2021). Children in this study expressed that effective communication involved good listening skills and the ability to understand non-verbal cues such as reading children’s body language. Other studies have identified that when children feel heard by adults, it fosters feeling of trust and facilitates their participation in decision-making (Sjöberg et al., 2015). But when adults speak over children, or health professionals respond to parents who speak for them, children feel excluded and disregarded and are denied a voice (Andersen and Dolva, 2015; Coyne and Gallagher, 2011). Despite the facilitating role of health professionals and parents in children having their views heard (Foster et al., 2022), adult attitudes to children’s views being included in their healthcare can still be paternalistic(Dickinson et al., 2014), and views that children lack capacity to make informed decisions still prevail (Traseira and Singh, 2020).

Children in this study reported that they were often given information in ways they could understand and were given time to think about that information and ask questions. This is recognised as a key enabling factor for inclusion in decision-making about their healthcare (Kilkelly and Savage, 2013; Lundberg et al., 2021; Quaye et al., 2021). While health professionals seemed to agree that being child friendly meant involving children in conversations and making sure they were well-informed, not all will have training or experience to do so. Interventions that focus on improving communication and informing children are needed and can help increase children’s involvement in consultations (Bray et al., 2018).

When children are involved in conversations and decisions, they feel part of a team and more ‘active’ in the process of their healthcare (Davies et al., 2024). This study identified that when children are included in multi-disciplinary rounds and clinical handovers, it builds their confidence and shared decision-making can be facilitated. Children’s descriptions of teamwork were similar to those of shared decision-making, a process where health professionals work in collaboration with patients to make care and treatment decisions (Coyne et al., 2014). Shared decision-making practices are associated with empowerment (Bjønness et al., 2020), better engagement in treatment (Bejarano et al., 2015), and increased satisfaction with care (Sleath et al., 2018). However, there are several obstacles to implementing shared decision-making (Hayes et al., 2018; Jordan et al., 2019; Wijngaarde et al., 2021) and further research on its effectiveness, including with children, is urgently needed (Cheng et al., 2017; Coyne et al., 2016b).

Children in this study clearly identified that to understand, communicate, and make decisions in healthcare, they required the support of their family. Children expressed the need of their parents to help them make decisions, especially when they feel overwhelmed. Parents play an important role in children being listened to and involved in healthcare decision-making (Kebbe et al., 2019; Kilkelly and Savage, 2013; Quaye et al., 2019). It is important to identify, however, that parents’ wishes can sometimes conflict with the wishes of their children, and parental needs can dominate medical consultations (Kebbe et al., 2019; Teleman et al., 2021). Parents have also been found to side with health professionals and not support children in challenging circumstances, and this can be a major obstacle to children being heard (Hallström and Elander, 2004).

## Limitations

While qualitative research does not aim to be generalisable, we acknowledge that findings of this study are based on responses of 20 children and young people in Sydney, Australia. The experiences of these children may not be representative of children in other countries. Children under the age of 7 years were excluded from this study, as inclusion of younger children would require different research and data collection methods, and this was outside of the scope of this study. Although unintentional, the eventual cohort of participants were mostly over 11 years (*n* = 18). It is therefore recognised that the experiences of younger children are not reflected in this study, and this should be an area of further research. It is also acknowledged that experiences of Aboriginal and Torres Strait Islander children, who were respectfully excluded from this study, are not represented and their experiences may significantly differ from experiences of non-indigenous children.

## Implications for practice and research

Findings from this inductive qualitative content analysis have relevance to practitioners working with children in a variety of healthcare settings. Children’s ability to have their views heard in healthcare is fundamental to delivery of child-centred care (Coyne et al., 2016a). Therefore, a focus on factors that influence children’s ability to express their views and to be heard is essential to child-centred care delivery. Factors identified in this study as facilitating this (time, relationships, communication, teamwork, and family support) are offered to inform child-centred practices and development of child-centred policies, procedures, and education programs. This, in turn, can help address persistent issues of children’s views not being heard or taken seriously, and exclusion of children from decision-making processes in healthcare. There is growing evidence of the importance of children being included in their healthcare and recommendations for practice. Despite this, children still struggle to have their views heard and taken seriously. Research focused on the translation of findings from this and other studies into practice is required. Further research that investigates the experiences of younger children, using methodologies that facilitate young children to express their views, is also urgently needed.

## Conclusion

The overall aim of this study was to understand children’s and young people’s experiences of expressing their views and having their views heard in healthcare. Through an inductive content analysis, factors that supported children to express their views and to have them heard were identified. These factors were time, relationships, communication, teamwork, and family support. The identification of these supporting factors can assist health professionals in the development of child-centred education, policy, and practice. The views of the children in this small qualitative study add to the existing literature on child rights in healthcare.
